# What are the roles of positive psychological construct in blended learning contexts? Integrating academic buoyancy into the Community of Inquiry framework

**DOI:** 10.3389/fpsyg.2024.1354156

**Published:** 2024-04-05

**Authors:** Yan Yang, Yoon Fah Lay

**Affiliations:** ^1^Department of College English, Zhejiang Yuexiu University, Shaoxing, China; ^2^Faculty of Psychology and Education, Universiti Malaysia Sabah, Kota Kinabalu, Sabah, Malaysia; ^3^Faculty of Social Sciences and Liberal Arts, UCSI University, Kuala Lumpur, Malaysia; ^4^School of Liberal Arts and Sciences, Taylor's University, Kuala Lumpur, Malaysia

**Keywords:** academic buoyancy, blended learning contexts, Community of Inquiry framework, teaching presence, social presence and cognitive presence

## Abstract

**Introduction:**

In the post-epidemic era, blended learning has become a social trend for the future of higher education, and scholars have endeavored to understand the factors that influence student learning in these blended communities. Communities of Inquiry is a conceptual framework that describes the components of blended learning environments, indicating teaching presence, social presence, and cognitive presence. However, the framework fails to adequately explore how individual learning motivational factors influence student learning. Therefore, this study extends the Community of Inquiry framework by drawing on a positive psychological construct-academic buoyancy to reveal the relationship between academic buoyancy and the three presences through empirical research.

**Methods:**

The theoretical model was validated by SPSS 26.0 and smartPLS4.0. To evaluate the measurement and structural models, structural equation modeling (SEM) was carried out using the partial least squares (PLS) method.

**Findings:**

(a) Teaching presence positively predicts academic buoyancy, and academic buoyancy positively predicts social presence and cognitive presence; (b) academic buoyancy mediates teaching presence and social presence, as well as teaching presence and cognitive presence; and (c) academic buoyancy acts as a chain mediator between teaching presence and cognitive presence through social presence.

**Discussion:**

The results of this study fill a gap in the multiple roles of individual positive psychological construct-academic buoyancy in blended learning communities, extend the Community of Inquiry theoretical framework, and provide empirical evidence for blended learning quality and practical improvement strategies.

## 1 Introduction

Innovations and developments in information technology have made more sustainable and evidence-based blended learning already a social trend for the future development of higher education (Liu et al., 2022). The Horizon Report 2022 (Teaching and Learning Edition) states that in the post-epidemic era, higher education should continue to optimize blended learning spaces and blended teaching models, and promote the normalization of the dual-line integration teaching model (EDUCAUSE, 2022). China's higher education authorities at all levels have also recognized a large number of first-class undergraduate online and offline blended courses at the national, provincial and municipal levels, which have been used as the basis for blended education in universities and colleges. Since then, blended learning has also become a mainstream learning approach in the post epidemic era (Huang and Gong, 2023). As an innovative product of the deep integration of information technology and education and teaching (Porter et al., 2014), blended learning reduces the drawbacks of emotional communication barriers, weak sense of presence, and poor “screen-to-screen” monitoring exposed in single-line education, and effectively realizes the complementary strengths of offline and online learning (Liu et al., 2022). Scholarship related to blended learning is growing rapidly as more and more courses adopt blended learning models. On one hand, researchers suggest students in blended learning value interaction with the instructor (Al Mamun et al., 2022; Wang et al., 2023), while others suggest blended learning works best when peer collaboration occurs (Sun et al., 2017; Al-Samarraie and Saeed, 2018; López-Pellisa et al., 2021). The Community of Inquiry framework (Garrison et al., 1999) combines these two perspectives and provides theoretical conceptualizations of how teaching presence and social presence in blended learning affect student learning, emphasizing the impact of external environmental factors on student learning in blended communities, but the individual learner factors have not been sufficiently explored and conceptualized (Shea and Bidjerano, 2010; Lan et al., 2018b). Moreover, in practice, scholars have found that problems related to individual psychological factors such as lack of motivation to learn, lack of willpower, and lack of ability to overcome academic difficulties and challenges have become the most significant barriers to success in blended learning for college students (Li, 2022; Podsiadlik, 2023). So, are learners' individual psychological factors an integral part of what drives student learning in a blended learning community?

The Community of Inquiry framework is an important theory in blended learning research in higher education. The theory suggests that effective blended learning relies on the organic synergy and interaction of three system elements: teaching presence, social presence and cognitive presence (Garrison et al., 1999). Research related to the three presences suggests that instructors (teaching presence) influence collaboration and learning (social presence and cognitive presence, respectively), and peer collaboration (social presence) influences student learning (cognitive presence; Garrison et al., 2010; Al-Saggaf and Rosli, 2021). The Community of Inquiry framework's most contemporary uses have focused on these three main presences (Cooper and Scriven, 2017; Yu and Li, 2022; Chimbo et al., 2023). However, with the depth of research, scholars have found that if the descriptive and explanatory power of the Community of Inquiry framework is to be enhanced, individual learner factors affecting learning should be added to the model, as individual factors directly affect the knowledge acquisition and cognitive engagement effects of students in the blended learning process (Shea and Bidjerano, 2010; Lan et al., 2018b). Some scholars have attempted to expand the framework in terms of individual positive psychological factors, and the results have shown that self-efficacy (Akcaoglu and Akcaoglu, 2022; Doo et al., 2023), self-regulation (Cho et al., 2017; Xue et al., 2023), motivation (Kilis and Yildirim, 2018; Zuo et al., 2022), and emotions (Espino et al., 2021; Sundgren et al., 2023) are related to the three presences in Community of Inquiry framework, but most of the above studies are limited to the theoretical level or online learning contexts, and it is still to be explored whether the individual-level positive psychological factors in blended learning contexts are related to the three presences in Community of Inquiry framework. Therefore, in this study, we hypothesize that personal-level positive psychological factors are a useful factor to extend the Community of Inquiry framework by attempting to more fully conceptualize the scope of presence in blended learning contexts. To this end, we rely on academic buoyancy, an individual psychological construct, to shed light on the broader components of individual-level success in blended learning environments.

Focusing on the positive aspects of personal success, academic buoyancy refers to a student's ability to successfully overcome difficulties and challenges encountered in daily academic life (Martin and Marsh, 2008). Theoretically, academic buoyancy is a noteworthy indicator of quality of learning, teacher satisfaction, student engagement, and overall self-confidence (Martin and Marsh, 2009; Martin, 2014). Practically, academic buoyancy partially mediates or fully mediates the relationship between teacher support (teaching presence) and collaborative learning engagement (social presence) and learning quality (Rohinsa et al., 2019; Granziera et al., 2022; Li et al., 2023). However, the above findings are from face-to-face traditional classroom or online environments, and it remains to be investigated whether they are applicable to blended learning environments. Based on this, the main objectives of this study are (a) to propose academic buoyancy as a type of presence within the Community of Inquiry framework in blended learning environments, and (b) to empirically investigate the relationship between academic buoyancy in blended learning environments and the three existing presences of the Community of Inquiry framework.

## 2 Literature review

### 2.1 Community of Inquiry framework

Based on years of blended learning practice, Canadian scholars such as Garrison et al. (1999) conceptualized presence in blended learning contexts and proposed the Community of Inquiry theoretical framework, which is a framework that highlights three key elements of presence in blended learning contexts: teaching presence, social presence and cognitive presence.

Teaching presence has three main functions: instructional design and organization, facilitating dialogue, and direct instruction. Instructors are responsible for designing curriculum that promotes cognitive presence and social presence (Garrison et al., 1999). Although teaching presence typically exists within a community of teachers, it can also extend to any learner in a community of inquiry (Garrison and Akyol, 2013; Rubio et al., 2018). And learners also play a key role in creating productive blended learning contexts. Social presence refers to the learner's ability to project “personal characteristics” onto the blended learning community and to express “true self” socially and emotionally (Garrison et al., 1999). It is understood as an individual's ability to “construct and validate meaning through critical, sustained dialogue and reflection” within a community (Garrison et al., 1999). It is manifested in all stages of learning, including triggering events (initiation of learning actions), exploration (information search), integration (synthesizing knowledge into a coherent idea) and resolution (problem solving; Garrison et al., 1999; Shea et al., 2012). These three elements interact and effectively collaborate to construct knowledge, facilitating a social constructivist form of blended learning contexts and creating the blended learning Community of Inquiry theoretical framework.

The Community of Inquiry framework originated from the blended learning experience (Garrison et al., 1999), and most of the previous studies exploring blended learning from the perspective of Community of Inquiry framework have either been limited to the construction of theoretical models (Shen and Sheng, 2015; Qiao, 2017), or to investigating the experience of using the model and the evaluation of perceptions (Lu et al., 2018; Wang and Liu, 2019), and there are few empirical studies that consider blended learning from the multidimensional variable perspective of the Community of Inquiry framework (Wu et al., 2017; Lan et al., 2020), especially in the context of EFL courses (He and Huang, 2023; Jia and Gao, 2023). In addition, while teaching presence, social presence, and cognitive presence are necessary elements for creating a blended learning context, the Community of Inquiry framework does not fully conceptualize how individual learner factors-positive psychological factors-influence student learning. In view of this, based on the blended learning context of an EFL course, academic buoyancy was incorporated into the community of inquiry model to explore the role of individual positive psychological factors in a blended learning community through empirical data.

### 2.2 Academic buoyancy

Psychologists Martin and Marsh (2008) first developed the concept of academic buoyancy from a positive psychology perspective, which refers to the ability of students to successfully overcome the difficulties and challenges they encounter in their daily academic lives. These challenges can range from poor academic performance, tight study schedules, exam pressure, and difficult classroom assignments. A similar concept to that of buoyancy is academic resilience, but the dilemmas faced by academic resilience refer to significant, long-term difficulties encountered by students, and its subjects usually refer to minority groups in special hardship situations, such as students in poverty, chronically low achievers, and students with poor learning abilities (Martin et al., 2010); whereas, academic buoyancy is targeted at all students, as difficulties and setbacks are unavoidable for students. Academic buoyancy focuses on an individual's strengths rather than weaknesses, and is considered a construct or state rather than a characteristic, meaning that it can be adjusted through training (Martin, 2013).

### 2.3 Academic buoyancy and Community of Inquiry framework

The Community of Inquiry framework considers the centrality of teacher roles (“teaching presence”), group dynamics (“social influence”), and student cognition (“cognitive presence”) in blended learning communities, but overemphasizes environmental factors in the learning process at the expense of the role inherent in the individual learner, making it overly reliant on standardized learning communities in its practical application (Stenbom et al., 2016). Given the highly participatory nature of blended learning, which relies heavily on student engagement (Al-Samarraie and Saeed, 2018), we propose to extend the Community of Inquiry framework by exploring the unique role of individual positive psychological factors (academic buoyancy) in blended learning environments. Similar to Community of Inquiry framework's cognitive presence, academic buoyancy recognizes the presence of the individual student. Cognitive presence is the degree to which learners acquire meaning construction and understanding, which cannot be achieved without the mental developmental process of higher-order thinking, and academic buoyancy represents positive psychological factors of mental development. Therefore, considering academic buoyancy as part of a broader Community of Inquiry framework not only contributes to a deeper understanding of metacognition in blended learning (Garrison and Akyol, 2015), but also allows scholars to realize the key role learners play in Community of Inquiry (Shea and Bidjerano, 2012). According to research at the intersection of motivation and pedagogical theories, when learners are confidently engaged in a learning community, they are largely dependent on individual-level motivators, and are more likely to achieve blended learning success by appropriately fostering these motivators (Nugroho et al., 2023). Therefore, this study integrates academic buoyancy into a blended learning environment to delve deeper into the personal-level factors that contribute to the success of the Community of Inquiry framework in order to facilitate the creation of thriving blended learning communities. Critical reflective dialogue and collaborative knowledge construction are critical to developing the metacognitive aspects of blended learning contexts, and individual factors determine participation and learning in blended communities (Sun et al., 2017; López-Pellisa et al., 2021). Academic buoyancy recognizes the key individual psychological factors required to develop Community of Inquiry framework, making it an appropriate presence at the individual level in blended learning environments.

In the Community of Inquiry framework, teaching presence serves as a conceptual anchor to describe the impact of teachers in blended environments through curriculum design, facilitated dialog, and direct instruction. It is considered a central organizing element of Community of Inquiry (Garrison and Akyol, 2013) and has a significant impact on student cognitive engagement, sense of community, and perceived learning outcomes (Garrison and Arbaugh, 2007). Prior evidence suggests that teaching presence is significantly and positively correlated with cognitive presence in blended learning (Law et al., 2019), that instructional design and organization, facilitated dialogue, and direct instruction are critical to the construction of student knowledge acquisition (Garrison and Cleveland-Innes, 2005), and that immediate feedback from teachers on student engagement in learning is effective in improving the quality of learning (Meech and Koehler, 2023). Related studies have also found that teaching presence in blended learning not only significantly affects cognitive presence, but also indirectly affects cognitive presence through learners' individual motivational factors as a mediating variable (Wu, 2017; Lan et al., 2018b). In recent years, as positive psychology research has flourished, scholars have begun to focus on the influence of teachers on students' positive psychological factors (e.g., academic buoyancy), such as teachers improve students' ability to effectively adapt to challenges and difficulties by maintaining close relationships with them (Yun et al., 2018). Established empirical studies have also shown that students' perceived teacher presence is predictive of their personality development (e.g., academic buoyancy), and can also indirectly affect students' academic buoyancy through cognitive and affective engagement (Chong et al., 2018; Granziera et al., 2022). Teacher support predicts the emergence of academic buoyancy, and academic buoyancy mediates the effect of teacher support on student engagement (Rohinsa et al., 2019). Furthermore, in English as a foreign language context, students' perceived teacher support can only indirectly influence educational outcomes through the full mediation of academic buoyancy (Li et al., 2023).

Social presence in a community of inquiry, which includes learners' ability to emotionally express themselves, communicate openly, and foster cohesion in the learning environment (Arbaugh and Benbunan-Fich, 2006; Garrison and Arbaugh, 2007), has strong correlations with both instructional effectiveness and student literacy (Bai et al., 2020; Sun and Yang, 2023). Social presence not only facilitates open communication, interpersonal interaction, and collaborative inquiry learning within a community, but also serves as a mediating variable between teaching presence and cognitive presence (Garrison et al., 2010), as it is related to both the teacher's responsibility (constructing and managing a learning community) in the teaching presence factor, as well as a prerequisite for students' development of cognitive presence (engaging in community learning activities). Thus, social presence is important in communities of inquiry (Garrison et al., 2010). Emotional expression in social presence is the foundation of a learning community of inquiry (Garrison and Akyol, 2013), open communication is the exchange of mutually courteous communication (Garrison et al., 1999), and group cohesion refers to the creation and maintenance of a sense of community through a sense of belonging (Garrison et al., 1999). High-quality emotional expression, open communication, and group cohesion require not only social interaction and interpersonal relationships, but also the creation of purposeful personal relationships (Garrison and Arbaugh, 2007). Specifically, the higher a student's level of buoyancy, the higher the behavioral engagement and emotional involvement associated with learning (Martin et al., 2017; Datu and Yang, 2018), which leads to a higher level of social presence throughout the learning community, and then a high level of social presence in turn contributes to an increase in community engagement and focus on success in a reciprocal manner. Therefore, students with high levels of buoyancy and resilience are more likely to experience higher levels of social presence in blended learning environments (Martin et al., 2017).

Cognitive presence is rooted in Dewey's model of practical inquiry (Garrison et al., 2001), which refers to the extent to which learners construct and validate meaning based on critical and sustained dialog and reflection (Garrison et al., 1999), and involves two dimensions, namely, critical-reflective dialog and collaborative knowledge construction. Individual learner factors (e.g., academic buoyancy) play a key role in cognitive engagement and knowledge acquisition, as learners with higher levels of ability to cope with academic challenges and frustrations engage more deeply in critical-reflective dialogues and collaborative constructive learning (Datu and Yang, 2018; af Ursin et al., 2021). Rather than passive recipients of information, students are social beings who learn through interaction, open discussion, application, and experience, and empowering students to engage in social learning environments becomes critical (Bryer and Seigler, 2012; Thomas and Allen, 2021). We see academic buoyancy as a means to improve the learning environment and, in turn, student learning. Therefore, students with higher levels of academic buoyancy may be better equipped to meet the challenges of blended learning courses (af Ursin et al., 2021) because they have a higher ability to cope with levels of academic difficulty and self-regulation, and are more likely to have a rich learning experience.

**Figure 1:**
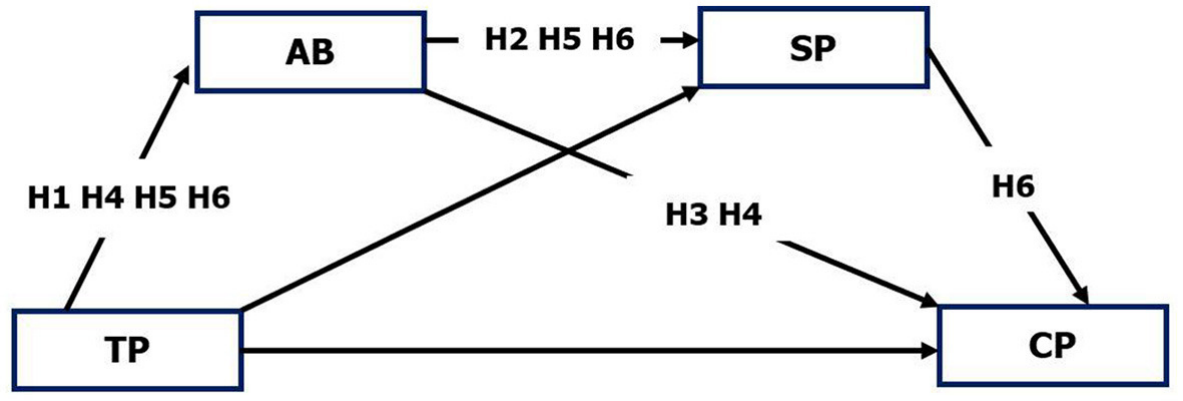
The proposed model. TP, Teaching Presence; AB, Academic Buoyancy; SP, Social Presence; CP, Cognitive Presence.

Based on the above research, this study incorporates academic buoyancy into the Community of Inquiry framework in order to extend past research and theoretically examine the multiple identities of academic buoyancy in a blended learning community of inquiry. As such, the following modeling hypotheses were proposed (shown in Figure 1).

Hypothesis 1: Teaching presence positively predicts academic buoyancy.
Hypothesis 2: Academic buoyancy positively predicts social presence.
Hypothesis 3: Academic buoyancy positively predicts cognitive presence.
Hypothesis 4: Academic buoyancy mediates teaching presence and cognitive presence.
Hypothesis 5: Academic buoyancy mediates teaching presence and social presence.
Hypothesis 6: Academic buoyancy acts as a chain mediator between teaching presence and cognitive presence through social presence.

## 3 Methodology

### 3.1 Participants

This study investigated undergraduate students with blended course learning experience in three universities in eastern China. The current study selected 78 students as pilot study participants to test the reliability and validity of the questionnaire. Subsequently, 312 students were selected through purposive sampling and random sampling, who took the online-offline blended first-class course in Zhejiang Province-English Country Profile (No. 689) led by the authors, as shown in Table 1. The course study started from September to December 2023, a total of 16 weeks, and all students had to complete 32 offline class periods and 12 online class periods as well as online resources.

**Table 1:** Demographic information of participants (*n* = 312).

**Gender**
Male (161)
Female (151)
**Year of college**
Junior (105)
Senior (207)
**College**
ZYU (143)
ZSU (94)
SUYC (75)
**Age**
20 (82)
21 (61)
22 (169)
**Field of study**
Chinese Literature (132)
Economics (83)
Big Data Management and Application (97)
**Experiencing years of blended learning**
1 (83)
1.5 (146)
2 (83)

### 3.2 Measures

#### 3.2.1 Instruments

Based on prior research, the variables were measured using an 11-point semantic differential scale (ranging from 0 = strongly disagree to 10 = strongly agree). The 11-point semantic difference scale allows for increased sensitivity, closer to the interval level of scaling and normality than the Likert 5- and 7-point scales (Leung, 2011), and better performance on unidimensionality and monotonicity (Hodge and Gillespie, 2007), and the survey was administered to intellectually able and sensitive college students, allowing for the use of the semantic differences scale to conduct self-administered scores (Oulo, 2017). The academic buoyancy scale used is the most widely used Martin and Marsh's (2008) “one-dimensional, four-item” scale. The scale consists of four items as shown in Table 2.

**Table 2:** Academic buoyancy scale.

**No**.	**Items**	**Reference**
1.	I'm good at dealing with setbacks (e.g., bad mark, negative feedback on my work).	Martin and Marsh, 2008
2.	I don't let study stress get on top of me.	
3.	I think I'm good at dealing with schoolwork pressures.	
4.	I don't let a bad mark affect my confidence.	

The Community of Inquiry (teaching presence, social presence and cognitive presence) scale uses the Chinese version of the Community of Inquiry Scale compiled by Lan et al. (2018a) using Chinese college students as the study sample, which consists of 27 items and uses the 11-point semantic differential scale, including 13 items of teaching presence, 5 items of social presence, and 9 items of cognitive presence. The Chinese version of the scale has high reliability, validity and structural rationality, and the internal consistency alpha coefficient of the scale is 0.955 (Lan et al., 2018a) as shown in Table 3.

**Table 3:** Community of Inquiry scale.

**No**.	**Items**	**References**
**Teaching presence**		
1.	The instructor clearly communicated important course topics.	Lan et al., 2018a												
2.	The instructor clearly communicated important course goals.
3.	The instructor provided clear instructions on how to participate in course learning activities.
4.	The instructor clearly communicated important due dates/time frames for learning activities.
5.	The instructor was helpful in identifying areas of agreement and disagreement on course topics that helped me to learn.
6.	The instructor was helpful in guiding the class toward understanding course topics in a way that helped me clarify my thinking.
7.	The instructor helped to keep course participants engaged and participating in productive dialogue.
8.	The instructor helped keep the course participants on task in a way that helped me to learn.
9.	The instructor encouraged course participants to explore new concepts in this course.
10.	Instructor actions reinforced the development of a sense of community among course participants.
11.	The instructor helped to focus discussion on relevant issues in a way that helped me to learn.
12.	The instructor provided feedback that helped me understand my strengths and weaknesses relative to the course's goals and objectives.
13.	The instructor provided feedback in a timely fashion.
**Social presence**		
1.	Online or web-based communication is an excellent medium for social interaction.	Lan et al., 2018a				
2.	I felt comfortable conversing through the online medium.
3.	I felt comfortable participating in the course discussions.
4.	I felt comfortable interacting with other course participants.
5.	I felt comfortable disagreeing with other course participants while still maintaining a sense of trust.
**Cognitive presence**		
1.	Problems posed increased my interest in course issues.	Lan et al., 2018a								
2.	Course activities piqued my curiosity.
3.	I felt motivated to explore content related questions.
4.	Brainstorming and finding relevant information helped me resolve content related questions.
5.	Online discussions were valuable in helping me appreciate different perspectives.
6.	Combing new information helped me answer questions raised in course activities.
7.	Learning activities helped me construct explanations/solutions.
8.	Reflection on course content and discussions helped me understand fundamental concepts in this class.
9.	I can apply the knowledge created in this course to my work or other non-class related activities.

#### 3.2.2 Pilot study

This pilot study used SPSS 26.0 for exploratory factor analysis (EFA) to improve the reliability and validity of the questionnaire and to remove unnecessary items. The specific criteria were as follows: sphericity Bartlett's test (*p* < 0.500), explained cumulative variance (≥50%), commonality (≥0.300), Kaiser-Meyer-Olkin test (>0.600), and eigenvalues (≥1.000; Barrett and Morgan, 2005; Hair et al., 2006; Pallant, 2011). Items with Cronbach's alpha lower than 0.700 were deleted (Hair et al., 2010). After testing, the preliminary findings showed that the questionnaire had good reliability and validity and all items met the above criteria. The results of each scale are shown in Table 4.

**Table 4:** Results of pilot study.

**Scale**	**Cronbach's alpha**	**KMO**	**Sphericity Bartlett test**	**Cumulative variance explained**	**The smallest items communalities**	**Eigenvalue**
Teaching presence	0.959	0.944	0.000	69%	0.581	≥1.00
Academic buoyancy	0.843	0.797	0.000	67%	0.520	≥1.00
Social presence	0.804	0.709	0.000	72%	0.702	≥1.00
Cognitive presence	0.911	0.885	0.000	65%	0.492	≥1.00

### 3.3 Data collection and data analysis

The questionnaires were distributed through Wenjuanxing-an online survey platform, and all students filled in the questionnaires after their teachers explained the contents of the questionnaires. A total of 330 questionnaires were returned in the formal survey (November 2023), with 312 valid questionnaires and a validity rate of 94.5%. Similar to Arbaugh (2007) and Garrison et al. (2010), we controlled for the age of the participants and the length of previous blended learning experiences they had engaged in. These controls were useful when examining differences in respondents' perceptions of each construct.

Data were analyzed using partial least squares (PLS), which was carried out through Smartpls 4, and outliers were removed prior to data analysis. In order to evaluate the measurement and structural models, a PLS approach was carried out for structural equation modeling (SEM; Hair et al., 2017). For hypothesis testing, a standard PLS algorithm was used to assess the significance level of the estimates on the basis of 5,000 bootstraps as suggested by Hair et al. (2011).

## 4 Findings

### 4.1 Descriptive statistics of variables

Descriptive statistics of the variables using SPSS 26.0 showed that the variables were at a moderate to high level of student teaching presence [TP, M (Mean) = 7.67, SD (Standard Deviation) =1.49], academic buoyancy (AB, M = 7.42, SD = 1.63), social presence (SP, M = 7.38, SD = 1.65), and cognitive presence (CP, M = 7.20, SD = 1.72).

### 4.2 Measurement model

**Figure 2:**
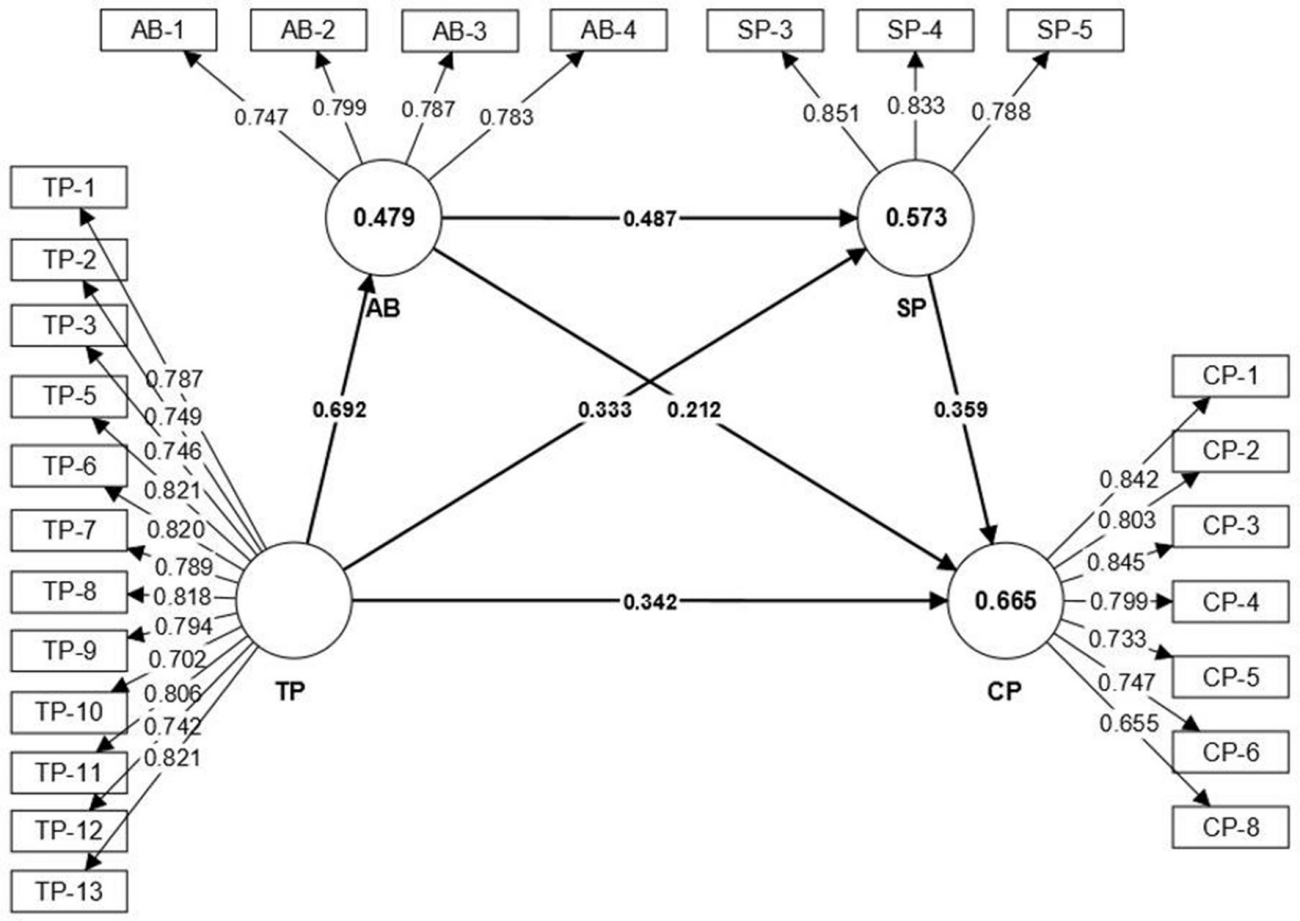
PLS-path analysis of path coefficients and *R*^2^ values (*n* = 312).

Following the recommendations of Hair et al. (2017), a two-step approach was used in this study. The first step is to test and assess the convergent validity and reliability. Convergent validity is obtained when the model meets the following criteria. Firstly, the loadings should be over 0.70 or higher (Hair et al., 2014), however, items below 0.70 should only be considered for removal from the weighing when removing them results in an increase in composite reliability, and items <0.40 should always be removed from the measurement construct (Hair et al., 2017). Second, composite reliability should exceed 0.70 (Gefen et al., 2000). Finally, Fornell and Larcker (1981) stated that the average variance extracted (AVE) should be more than 0.50. Therefore, according to the results, after removing some items with loading lower than 0.70, the model fulfilled all the above criteria, and although the CP-8 in social presence was lower than 0.70, the composite reliability was reduced by removing it, so finally it was given to be retained as shown in Table 5, Figure 2.

**Table 5:** Evaluation of measurement model.

**Variable**	**Cronbach's alpha**	**rho_A**	**Composite reliability**	**Average variance extracted (AVE)**
Teaching presence	0.943	0.949	0.950	0.614
Academic buoyancy	0.799	0.855	0.861	0.608
Social presence	0.768	0.788	0.864	0.680
Cognitive presence	0.890	0.898	0.914	0.604

### 4.3 Discriminant validity

In the follow-up phase, we used the Heterotrait-Monotrait Ratio (HTMT) criterion proposed by Henseler et al. (2016) to assess discriminant validity. As suggested by Kline (2011), discriminant validity is considered established when the value is below the 0.90 threshold. In our research model, the HTMT values ranged from 0.675 to 0.877, indicating that discriminant validity was satisfied. The evaluations conducted validated the convergent validity, reliability, and discriminant validity of the model.

### 4.4 Structural model

In order to test these hypotheses, the structural model was first assessed for covariance and all predictor constructs met the criteria, i.e., the variance inflation factor (VIF) ranged between 1.243 and 2.968, which is >1 and much <5, indicating very satisfactory reliability (Hair et al., 2017). Therefore, the results do not indicate multicollinearity issues and support formability. The weight of each size was above the recommended value of 0.10 (as shown in Figure 2; Hair et al., 2017). Secondly, a bootstrapping procedure was used with a resampling rate of 5,000 as suggested by Hair et al. (2017), which resulted in Beta, *p*-values, *t*-values and bootstrap confidence intervals. This analysis used the thresholds of one-tailed *t*-test and the results were 1.645 (significance level < 0.05), 2.327 (significance level < 0.01), and 3.092 (significance level < 0.001) as described by Hair et al. (2017), according to the bootstrap process, we can find that the standardized path coefficients for TP -> AB, AB -> SP, AB -> CP, TP -> AB -> CP, TP -> AB -> SP and TP -> AB -> SP -> CP are all positive with 0.692, 0.487, 0.212, 0.147, 0.337, and 0.121, as shown in Table 6, Figure 2, which indicates a positive relationship between the variables. The standardized path coefficients of TP -> AB and AB-> SP reached 0.692 and 0.487, indicating that the former could positively predict the latter to a large extent. Therefore, hypotheses H1–H6 are supported.

**Table 6:** Assessment of structural model (*n* = 312).

**Relationship**	**Standard path coefficients**	**Sample mean (M)**	**Standard deviation**	***T*-statistics**	***P*-values**	**Results**
TP -> AB	0.692^***^	0.694	0.024	28.448	0.000	H1 supported
AB -> SP	0.487^***^	0.485	0.045	10.876	0.000	H2 supported
AB -> CP	0.212^***^	0.212	0.042	5.035	0.000	H3 supported
TP -> AB -> CP	0.147^***^	0.148	0.030	4.877	0.000	H4 supported
TP -> AB -> SP	0.337^***^	0.336	0.028	11.956	0.000	H5 supported
TP -> AB -> SP -> CP	0.121^***^	0.121	0.021	5.679	0.000	H6 supported

^***^p < 0.001, *t* > 3.092 (one tailed).

### 4.5 *R^2^* value and *Q^2^* value

The coefficient of determination (*R*^2^) measures the predictive accuracy of the model and is determined by the squared correlation between the actual and predicted values of a particular endogenous construct or dependent variable (Hair et al., 2016). *R*^2^ can take a range of values from 0 to 1, with higher values denoting higher predictive accuracy. A strong *R*^2^ value is considered to be 0.75, moderate 0.50 and weak 0.25 (Hair et al., 2016). In this study, the *R*^2^ results were academic buoyancy = 0.479, social presence = 0.573, and cognitive presence = 0.665 (as shown in Figure 2). This shows that the data in this study have good prediction accuracy.

As noted by Stone (1974), *Q*^2^ is a criterion for predictive relevance. Henseler and Fassott (2009) also highlighted its utility in assessing the predictive ability of research models. *Q*^2^ utilizes a blindfold procedure to assess the predictive validity of a model by partial least squares (PLS). *Q*^2^ values above zero indicate that the exogenous construct is predictively relevant to the endogenous one. 0.02 is considered weak, 0.15 is considered moderate, and 0.35 is considered strong (Hair et al., 2011). In the present study, the *Q*^2^ results (academic buoyancy = 0.229; social presence = 0.372; cognitive presence = 0.394) indicate that the research model has excellent predictive relevance.

## 5 Discussion

The growing importance of blended learning in higher education has prompted researchers to more fully understand how phenomena emerge and influence student learning in the blended learning contexts. While the Community of Inquiry framework effectively conceptualizes the teaching, social and cognitive aspects of online environments, there is still a lack of research detailing the role of individual-level motivators associated with Community of Inquiry elements. While there have been studies (Wu et al., 2017; Lan et al., 2018b, 2020) that have attempted to come to grips with this gap by emphasizing the importance of learner-oriented presence in Community of Inquiry, they have either been limited to online environments or at the theoretical level. Building on their work, we looked for support for increasing students' motivational states to extend Community of Inquiry through empirical research to further recognize the role of the individual. To this end, we explored the links between academic buoyancy and the three presences in the Community of Inquiry framework.

Our findings suggest that academic buoyancy plays an important role within the Community of Inquiry framework. Firstly, this finding further supports that teaching presence is considered a core organizing element of Community of Inquiry framework (Garrison and Akyol, 2013) and has a significant impact on sense of community (Garrison and Arbaugh, 2007). Yun et al.'s (2018) study backs up the conclusion that teaching presence positively predicts academic buoyancy. They noted that teachers improve students' ability to effectively adapt to challenges and difficulties by maintaining a close relationship with them. Students' perceived teacher presence is predictive of their personality development (e.g., academic buoyancy). Research by Chong et al. (2018), Rohinsa et al. (2019), and Granziera et al. (2022) also suggests that teacher support predicts the emergence of academic buoyancy. Our study affirms the significant impact of teachers in blended learning environments, emphasizing the critical role of teachers in this regard. Through effective instruction and thoughtful curriculum design, teachers have a significant and direct impact on all aspects of student learning, including cognitive presence, social interactions among students, and the degree to which students are motivated to face every day academic challenges. In essence, these results push the boundaries of the Community of Inquiry framework and expand our understanding of its scope. Second, our study emphasizes that academic buoyancy influences social presence in blended learning environments. Essentially, students with the ability to actively cope with academic difficulties and challenges are more likely to actively participate in collaborative exchanges within the medium of a blended course. These results extend existing research on the impact of academic buoyancy on knowledge exchange in learning communities by demonstrating a similar relationship within the unique context of blended learning (Thomas and Allen, 2021). Once again, academic buoyancy positively predicted cognitive presence, which is consistent with previous studies showing that individual learner factors are predictors of cognitive presence (Lan et al., 2018b, 2020). And our findings further confirm that positive psychological factors (academic buoyancy) have a similar positive effect on student learning in a blended course context.

In addition, current research has proved that students' academic buoyancy mediates teaching and cognitive presence as well as teaching and social presence, and that academic buoyancy acts as a chain mediator between teaching and cognitive presence through social presence. These findings are validated by previous similar studies that teaching presence indirectly influences cognitive presence through the mediating variable of individual learner factors (Lan et al., 2018b). Teacher support indirectly influences student engagement (Chong et al., 2018; Rohinsa et al., 2019) and educational outcomes (Li et al., 2023) through the mediation of academic buoyancy. The current study fills a gap in the literature on the intrinsic relationship between academic buoyancy and the three presences in the Community of Inquiry framework in blended learning contexts. It explains the critical role of positive psychological factors of individual students in blended learning communities in open communication, knowledge construction and deep knowledge acquisition. Attempts to improve teachers' course organization and design, learners' psychological level of coping with academic difficulties and challenges, and the quality of collaborative inquiry learning in learning communities are effective measures to improve students' future blended learning outcomes.

In the post-epidemic era, blended learning is becoming the norm for university students. However, a persistent need to improve the quality of blended learning remains (Ellis et al., 2016; Han and Ellis, 2019). This study incorporates academic buoyancy into the Community of Inquiry framework as a mediating variable based on previous research emphasizing individual student factors, revealing the important role of academic buoyancy in blended learning contexts, which provides important insights into the improvement of blended learning quality. In the process of blended learning contexts, teachers should design effective learning activities (teaching presence), create a good learning atmosphere (social presence), give learners adequate academic guidance, adequate emotional care or after-school services, pay attention to cultivating their positive personalities, enhance their academic buoyancy level, make it easier for them to adapt to the challenges and difficulties of blended learning, and provide an external environment for realizing high levels of cognitive activity and internal psychology, i.e., teachers should simultaneously coordinate the relationship between teaching presence, social presence and cognitive presence, design effective learning activities, establish a good learning environment, and at the same time actively enhance students' level of academic buoyancy to ensure that cognitive stimulation occurs so as to optimize students' learning to the greatest extent possible. At the same time, teaching administrators should incorporate the enhancement of learners' academic buoyancy level into the top-level design of the blended course system, and at the same time incorporate academic buoyancy content into the university's supporting psychological training courses for college students, so as to enhance their academic buoyancy level, and to help them to achieve the success of blended learning. In addition, as an individual learner, he or she should actively participate in blended learning activities, conscientiously complete the online and offline learning tasks, and through the interaction with learning peers, teachers and learning resources, enhance the interest in online learning and enthusiasm for learning, which in turn increases the durability of blended learning.

## 6 Limitations and implications

Whilst this study contributes by recognizing the inclusion of academic buoyancy in Community of Inquiry and investigating its association with correlational presences, it is important to acknowledge its limitations. Academic buoyancy predominantly encompasses positive individual-level states, and we recognize the need for a more comprehensive conceptualization of learning presence that considers both positive and negative psychological states. By integrating the assessment of negative psychological traits (Sharma and Sarkar, 2020; Sumarsono et al., 2021), scholars can provide a more nuanced examination of the interrelationships between learning presence and other presences within the Community of Inquiry.

Second, in terms of sample selection, although this study conducted purposive cluster-based sampling and randomized cluster sampling on three universities in eastern China, it did not sample and survey universities in other regions. Therefore, it is recommended that future research conduct comparative studies by sampling and surveying universities in other regions of China or universities in other countries. Parallel longitudinal studies can also help to better understand the impact of blended learning on learners from different regions in China or other countries. In addition, future research could investigate the impact of different variables, such as the influence of students' gender and specialization (field of study) on students' perspectives.

Third, this study relied on a single questionnaire administered to students. The lack of multiple, lagged assessments limits the extent to which results can be causally interpreted. While advanced structural modeling techniques provided valuable insights, we expect that future research will causally assess relationships within the Community of Inquiry framework, thus providing deeper insights into the dynamic interactions between its components.

## Data availability statement

The original contributions presented in the study are included in the article/supplementary material, further inquiries can be directed to the corresponding author.

## Ethics statement

The studies involving humans were approved by Faculty of Psychology and Education, Universiti Malaysia Sabah. The studies were conducted in accordance with the local legislation and institutional requirements. The participants provided their written informed consent to participate in this study.

## Author contributions

YY: Writing – original draft. YL: Writing – review & editing.

## References

[B1] af Ursin P. Järvinen T. Pihlaja P. (2021). The role of academic buoyancy and social support in mediating associations between academic stress and school engagement in Finnish primary school children. Scand. J. Educ. Res. 65, 661–675. 10.1080/00313831.2020.1739135

[B2] Akcaoglu M. Akcaoglu M. O. (2022). Understanding the relationship among self-efficacy, utility value, and the community of inquiry framework in preservice teacher education. Int. Rev. Res. Open Distribut. Learn. 23, 86–106. 10.19173/irrodl.v23i1.5717

[B3] Al Mamun M. A. Lawrie G. Wright T. (2022). Exploration of learner-content interactions and learning approaches: the role of guided inquiry in the self-directed online environments. Comput. Educ. 178:104398. 10.1016/j.compedu.2021.104398

[B4] Al-Saggaf M. A. Rosli A. S. (2021). The level of community of inquiry (CoI) presences in online classes among MSU BTESL students. TESOL Technol. Stud. 2, 65–78. 10.48185/tts.v2i1.175

[B5] Al-Samarraie H. Saeed N. (2018). A systematic review of cloud computing tools for collaborative learning: opportunities and challenges to the blended-learning environment. Comput. Educ. 124, 77–91. 10.1016/j.compedu.2018.05.016

[B6] Arbaugh J. B. (2007). An empirical verification of the community of inquiry framework. J. Asynchr. Learn. Netw. 11, 73–85. 10.24059/olj.v11i1.1738

[B7] Arbaugh J. B. Benbunan-Fich R. (2006). An investigation of epistemological and social dimensions of teaching in online learning environments. Acad. Manag. Learn. Educ. 5, 435–447. 10.5465/amle.2006.23473204

[B8] Bai X. M. Ma H. L. Zhao M. (2020). Influential mechanism of social presence on cognitive presence in community of inquiry. Modern Dist. Educ. Res. 6, 87–93. 10.3969/j.issn.1009-5195.2020.06.011

[B9] Barrett K. C. Morgan Jr. G. A. (2005). SPSS for Intermediate Statistics: Use and Interpretation. London: Psychology Press.

[B10] Bryer T. A. Seigler D. (2012). Theoretical and instrumental rationales of student empowerment through social and web-based technologies. J. Publ. Affairs Educ. 18, 429–448. 10.1080/15236803.2012.12001693

[B11] Chimbo B. Mutezo A. T. Maré S. (2023). Postgraduate students online learning challenges during COVID-19 within the CoI framework context. Cogent Educ. 10:2254673. 10.1080/2331186X.2023.2254673

[B12] Cho M. H. Kim Y. Choi D. (2017). The effect of self-regulated learning on college students' perceptions of community of inquiry and affective outcomes in online learning. Internet High. Educ. 34, 10–17. 10.1016/j.iheduc.2017.04.001

[B13] Chong W. H. Liem G. A. D. Huan V. S. Kit P. L. Ang R. P. (2018). Student perceptions of self-efficacy and teacher support for learning in fostering youth competencies: roles of affective and cognitive engagement. J. Adolesc. 68, 1–11. 10.1016/j.adolescence.2018.07.002 29986166

[B14] Cooper T. Scriven R. (2017). Communities of inquiry in curriculum approach to online learning: strengths and limitations in context. Austral. J. Educ. Technol. 33:3026. 10.14742/ajet.3026

[B15] Datu J. A. D. Yang W. (2018). Psychometric validity and gender invariance of the academic buoyancy scale in the Philippines: a construct validation approach. J. Psychoeduc. Assess. 36, 278–283. 10.1177/0734282916674423

[B16] Doo M. Y. Bonk C. J. Heo H. (2023). Examinations of the relationships between self-efficacy, self-regulation, teaching, cognitive presences, and learning engagement during COVID-19. Educ. Technol. Res. Dev. 3, 1–24. 10.1007/s11423-023-10187-3 36743449 PMC9885918

[B17] EDUCAUSE (2022). EDUCAUSE Horizon Report. (Teaching and Learning Edition). [EB/OL]. (2022-04-18) [2022-10-03]. Available online at: https://library.educause.edu/resources/2022/4/2022-educause-horizon-report-teaching-and-learning-edition (accessed April 18, 2022).

[B18] Ellis R. A. Pardo A. Han F. (2016). Quality in blended learning environments-significant differences in how students approach learning collaborations. Comput. Educ. 102, 90–102. 10.1016/j.compedu.2016.07.006

[B19] Espino D. P. Wright T. Brown V. M. Mbasu Z. Sweeney M. Lee S. B. (2021). “Student emotions in the shift to online learning during the COVID-19 pandemic,” in Advances in Quantitative Ethnography. ICQE 2021. Communications in Computer and Information Science, vol. 1312, eds. A. R. Ruis and S. B. Lee (Cham: Springer), 23.

[B20] Fornell C. Larcker D. F. (1981). Structural equation models with unobservable variables and measurement error: algebra and statistics. J. Market. Res. 18, 382–388. 10.1177/002224378101800313

[B21] Garrison D. R. Akyol Z. (2013). The community of inquiry theoretical framework. Handb. Dist. Educ. 3, 104–120. 10.4324/9780203803738.ch7

[B22] Garrison D. R. Akyol Z. (2015). Toward the development of a metacognition construct for communities of inquiry. Internet High. Educ. 24, 66–71. 10.1016/j.iheduc.2014.10.001

[B23] Garrison D. R. Anderson T. Archer W. (1999). Critical inquiry in a text-based environment: computer conferencing in higher education. Internet High. Educ. 2, 87–105. 10.1016/S1096-7516(00)00016-6

[B24] Garrison D. R. Anderson T. Archer W. (2001). Critical thinking, cognitive presence, and computer conferencing in distance education. Am. J. Dist. Educ. 15, 7–23. 10.1080/08923640109527071

[B25] Garrison D. R. Arbaugh J. B. (2007). Researching the community of inquiry framework: review, issues, and future directions. Internet High. Educ. 10, 157–172. 10.1016/j.iheduc.2007.04.001

[B26] Garrison D. R. Cleveland-Innes M. (2005). Facilitating cognitive presence in online learning: interaction is not enough. Am. J. Dist. Educ. 19, 133–148. 10.1207/s15389286ajde1903_2

[B27] Garrison D. R. Cleveland-Innes M. Fung T. S. (2010). Exploring causal relationships among teaching, cognitive and social presence: student perceptions of the community of inquiry framework. Internet High. Educ. 13, 31–36. 10.1016/j.iheduc.2009.10.002

[B28] Gefen D. Straub D. W. Boudreau M. C. (2000). Structural equation modelling and regression: guidelines for research practice. Commun. Associat. Inform. Syst. 4, 1–79. 10.17705/1CAIS.00407

[B29] Granziera H. Liem G. A. D. Chong W. H. Martin A. J. Collie R. J. Bishop M.. (2022). The role of teachers' instrumental and emotional support in students' academic buoyancy, engagement, and academic skills: a study of high school and elementary school students in different national contexts. Learn. Instr. 80:101619. 10.1016/j.learninstruc.2022.101619

[B30] Hair J. F. Anderson R. E. Babin B. J. (2010). Multivariate Data Analysis: A Global Perspective, 7th ed. Hoboken, NJ: Pearson Prentice Hall.

[B31] Hair J. F. Hult G. T. M. Ringle C. M. (2016). A Primer on Partial Least Squares Structural Equation Modeling (PLS-SEM). London: Sage publications.

[B32] Hair J. F. Hult G. T. M. Ringle C. M. Sarstedt M. (2017). A Primer on Partial Least Squares Structural Equation Modelling (PLS-SEM), 2nd Edn. New York, NY: SAGE Publication.

[B33] Hair J. F. Ringle C. M. Sarstedt M. (2011). PLS-SEM: indeed a silver bullet. J. Market. Theory Practice 19, 139–152. 10.2753/MTP1069-6679190202

[B34] Hair J. F. Sarstedt M. Hopkins L. G. Kuppel wieser V. (2014). Partial least squares structural equation modeling (PLS-SEM) an emerging tool in business research. Eur. Bus. Rev. 26, 106–121. 10.1108/EBR-10-2013-0128

[B35] Hair J. S. Black W. C. Babin B. J. Anderson R. E. Tatham R. L. (2006). Multivariate Data Analysis. Hoboken, NJ: Prentice-Hall.

[B36] Han F. Ellis R. A. (2019). Identifying consistent patterns of quality learning discussions in blended learning. Internet High. Educ. 40, 12–19. 10.1016/j.iheduc.2018.09.002

[B37] He Y. Y. Huang Y. Y. (2023). The relationship between metacognition and community of inquiry models - based on blended teaching practices in college english. J. Kunming Metallurgy College. 1, 78–86. 10.3969/j.issn.1009-0479.2023.01.013

[B38] Henseler J. Fassott G. (2009). “Testing moderating effects in PLS path models: an illustration of available procedures,” in Handbook of Partial Least Squares: Concept Methods and Applications, eds Esposito Vinzi V. Chin W. W. Henseler J. Wang H. (Berlin: Springer), 31.

[B39] Henseler J. Ringle C. M. Sarstedt M. (2016). Testing measurement invariance of composites using partial least squares. Int. Market. Rev. 33, 405–431. 10.1108/IMR-09-2014-0304

[B40] Hodge D. R. Gillespie D. F. (2007). Phrase completion scales: a better measurement approach than Likert scales? J. Soc. Serv. Res. 34, 1–12. 10.1300/J079v33n04_01

[B41] Huang Y. T. Gong Y. X. (2023). How deep learning occurs for undergraduate students in hybrid courses: an examination based on the pedagogical-social-technological triangle. J. Soochow Univ. 3, 107–118. 10.19563/j.cnki.sdjk.2023.03.009

[B42] Jia W. Gao X. Y. (2023). A study of the community of inquiry model for blended learning in university English—an analysis of the mediating effects of emotional presence and social presence. Foreign Language World 4, 82–90.

[B43] Kilis S. Yildirim Z. (2018). Investigation of community of inquiry framework in regard to self-regulation, metacognition and motivation. Comput. Educ. 126, 53–64. 10.1016/j.compedu.2018.06.032

[B44] Kline R. B. (2011). Principles and Practice of Structural Equation Modelling. New York, NY: Guilford Press.

[B45] Lan G. S. H. Zhong Q. J. Guo Q. Kong X. K. (2020). Research on the relationship between self-efficacy, self-regulated learning and community of inquiry model-based on blended learning in online learning space. China Educ. Technol. 12, 44–54.

[B46] Lan G. S. H. Zhong Q. J. Lv C. J. Song Y. T. (2018b). Exploring relationships between learning presence and community of inquiry model. Open Educ. Res. 5, 92−107. 10.13966/j.cnki.kfjyyj.2018.05.011

[B47] Lan G. S. H. Zhong Q. J. Lv C. J. Song Y. T. Wei J. C. (2018a). Construction of a Chinese version of the community of inquiry measurement instrument. Open Educ. Res. 3, 68−76. 10.13966/j.cnki.kfjyyj.2018.03.008

[B48] Law K. M. Geng S. Li T. (2019). Student enrollment, motivation and learning performance in a blended learning environment: the mediating effects of social, teaching, and cognitive presence. Comput. Educ. 136, 1–12. 10.1016/j.compedu.2019.02.021

[B49] Leung S. O. (2011). A comparison of psychometric properties and normality in 4-, 5-, 6-, and 11-point Likert scales. J. Soc. Serv. Res. 37, 412–421. 10.1080/01488376.2011.580697

[B50] Li X. Duan S. Liu H. (2023). Unveiling the predictive effect of students' perceived EFL teacher support on academic achievement: the mediating role of academic buoyancy. Sustainability 15:10205. 10.3390/su151310205

[B51] Li Y. (2022). Analysis of psychological disorders and adaptive influence of blended learning of college students. Comput. Intell. Neurosci. 2022:5418738. 10.1155/2022/5418738 36248951 PMC9553445

[B52] Liu X. F. Lan G. S. H. Wei J. C. Liu G. N. (2022). Digital transformation of education promotes the future development of higher education and teaching: macro trends, technologies and practices, and future scenarios-key points and considerations of the 2022 EDUCAUSE Horizon Report (Teaching and Learning Edition). J. Soochow Univ. 2, 115–128. 10.19563/j.cnki.sdjk.2022.02.010

[B53] López-Pellisa T. Rotger N. Rodríguez-Gallego F. (2021). Collaborative writing at work: peer feedback in a blended learning environment. Educ. Inform. Technol. 26, 1293–1310. 10.1007/s10639-020-10312-2

[B54] Lu D. Jie Y. G. Tang Y. W. Yang X. (2018). Research on a new blended learning mode oriented by critical thinking-taking english writing teaching as an example. China Educ. Technol. 6, 135–140.

[B55] Martin A. J. (2013). Academic buoyancy and academic resilience: exploring “everyday” and “classic” resilience in the face of academic adversity. Sch. Psychol. Int. 34, 488–500. 10.1177/0143034312472759

[B56] Martin A. J. (2014). Academic buoyancy and academic outcomes: towards a further understanding of students with attention-deficit/hyperactivity disorder (ADHD), students without ADHD, and academic buoyancy itself. Br. J. Educ. Psychol. 84, 86–107. 10.1111/bjep.12007 24547755

[B57] Martin A. J. Colmar S. H. Davey L. A. Marsh H. W. (2010). Longitudinal modelling of academic buoyancy and motivation: do the 5Cs hold up over time? Br. J. Educ. Psychol. 80, 473–496. 10.1348/000709910X486376 20170601

[B58] Martin A. J. Marsh H. W. (2008). Academic buoyancy: towards an understanding of students' everyday academic resilience. J. Sch. Psychol. 46, 53–83. 10.1016/j.jsp.2007.01.002 19083351

[B59] Martin A. J. Marsh H. W. (2009). Academic resilience and academic buoyancy: multidimensional and hierarchical conceptual framing of causes, correlates and cognate constructs. Oxf. Rev. Educ. 35, 353–370. 10.1080/03054980902934639

[B60] Martin A. J. Yu K. Ginns P. Papworth B. (2017). Young people's academic buoyancy and adaptability: a cross-cultural comparison of China with North America and the United Kingdom. Educ. Psychol. 37, 930–946. 10.1080/01443410.2016.1202904

[B61] Meech S. Koehler A. A. (2023). Instructor leadership and the community of inquiry framework: applying leadership theory to higher education online learning. Int. Rev. Res. Open Distribut. Learn. 24, 118–137. 10.19173/irrodl.v24i2.6953

[B62] Nugroho E. P. Hidayat K. Nurdin E. A. (2023). Development of E-learning-based blended learning to increase student learning motivation during a pandemic. APTISI Trans. Manag. 7, 160–169. 10.33050/atm.v7i2.1992

[B63] Oulo E. V. (2017). University students' attitudes as measured by the semantic differental. JSTOR 60, 152–158. 10.1080/00220671.1966.10883462

[B64] Pallant J. (2011). SPSS Survival Manual: A Step by Step Guide to data Analysis Using SPSS, 4th Edn. London: Allen and Unwin.

[B65] Podsiadlik A. (2023). The blended learning experiences of students with specific learning difficulties: a qualitative case study located in one British higher education institution. Int. J. Disabil. Dev. Educ. 70, 366–381. 10.1080/1034912X.2021.1876217

[B66] Porter W. W. Graham C. R. Spring K. A. Welch K. R. (2014). Blended learning in higher education: institutional adoption and implementation. Comput. Educ. 75, 185–195. 10.1016/j.compedu.2014.02.011

[B67] Qiao H. J. (2017). Research on blended english learning model based on inquiry society system. Technol. Enhanc. For. Lang. 4, 43–48.

[B68] Rohinsa M. Cahyadi S. Djunaidi A. Iskandar T. Z. (2019). The role of teacher support in predicting engagement through academic buoyancy. Int. J. Innov. Creat. Change 10:200–13.

[B69] Rubio F. Thomas J. M. Li Q. (2018). The role of teaching presence and student participation in Spanish blended courses. Comput. Assist. Lang. Learn. 31, 226–250. 10.1080/09588221.2017.1372481

[B70] Sharma S. Sarkar P. (2020). Efficiency of blended learning in reduction of anxiety: with special reference to high school students. Int. J. Grid Distribut. Comput. 13, 277–285.

[B71] Shea P. Bidjerano T. (2010). Learning presence: towards a theory of self-efficacy, self-regulation, and the development of a communities of inquiry in online and blended learning environments. Comput. Educ. 55, 1721–1731. 10.1016/j.compedu.2010.07.017

[B72] Shea P. Bidjerano T. (2012). Learning presence as a moderator in the community of inquiry model. Comput. Educ. 59, 316–326. 10.1016/j.compedu.2012.01.011

[B73] Shea P. Hayes S. Smith S. U. Vickers J. Bidjerano T. Pickett A.. (2012). Learning presence: additional research on a new conceptual element within the Community of Inquiry (CoI) framework. Internet High. Educ. 15, 89–95. 10.1016/j.iheduc.2011.08.002

[B74] Shen Y. Sheng Y. D. (2015). College english flipped classroom teaching based on inquiry community system. For. Lang. World 4, 81–89.

[B75] Stenbom S. Jansson M. Hulkko A. (2016). Revising the community of inquiry framework for the analysis of one-to-one online learning relationships. Int. Rev. Res. Open Distribut. Learn. 17, 36–53. 10.19173/irrodl.v17i3.2068

[B76] Stone M. (1974). Cross-validatory choice and assessment of statistical predictions. J. Royal Stat. Soc. 36, 111–133. 10.1111/j.2517-6161.1974.tb00994.x

[B77] Sumarsono D. Haryadi H. Bagis A. K. (2021). When blended learning is forced in the amid of COVID-19: what happen on EFL learners' speaking anxiety? J. Lang. Lang. Teach. 9, 305–315. 10.33394/jollt.v9i3.3906

[B78] Sun Z. Liu R. Luo L. Wu M. Shi C. (2017). Exploring collaborative learning effect in blended learning environments. J. Comput. Assist. Learn. 33, 575–587. 10.1111/jcal.12201

[B79] Sun Z. Yang Y. (2023). The mediating role of learner empowerment in the relationship between the community of inquiry and online learning outcomes. Internet High. Educ. 58:100911. 10.1016/j.iheduc.2023.100911

[B80] Sundgren M. Jaldemark J. Cleveland-Innes M. (2023). Disciplinary differences and emotional presence in communities of inquiry: teachers' expressions of digital technology-enabled teaching. Comput. Educ. Open 4:100134. 10.1016/j.caeo.2023.100134

[B81] Thomas C. L. Allen K. (2021). Driving engagement: investigating the influence of emotional intelligence and academic buoyancy on student engagement. J. Further High. Educ. 45, 107–119. 10.1080/0309877X.2020.1741520

[B82] Wang X. Sazalli N. Adnan W. N. A. W. (2023). Blended instructional strategies based on community of inquiry framework: a systematic review of the literature. Int. J. Adult Educ. Technol. 14, 1–20. 10.4018/IJAET.332812

[B83] Wang Y. K. Liu B. (2019). A practical study of online-offline mixed teaching of academic english for postgraduates. For. Lang. Teach. 5, 10–19. 10.13458/j.cnki.flatt.004615

[B84] Wu X. E. Chen X. H. Wu J. (2017). On the effects of presence on online learning performance. Mod. Distance Educ. 8, 66–73. 10.13927/j.cnki.yuan.2017.0014

[B85] Wu Y. J. (2017). Research on influential factors and its measurement of learners' online deep learning. E-educ. Res. 38, 57–63. 10.13811/j.cnki.eer.2017.09.008

[B86] Xue J. Xu X. Wu Y. Hu P. (2023). Student perceptions of the community of inquiry framework and satisfaction: examining the role of academic emotion and self-regulation in a structural model. Front. Educ. 8:1046737. 10.3389/feduc.2023.1046737

[B87] Yu Z. Li M. (2022). A bibliometric analysis of Community of Inquiry in online learning contexts over twenty-five years. Educ. Inform. Technol. 27, 11669–11688. 10.1007/s10639-022-11081-w 35610978 PMC9118826

[B88] Yun S. Hiver P. Al-Hoorie A. H. (2018). Academic buoyancy: exploring learners' everyday resilience in the language classroom. Stud. Sec. Lang. Acquisit. 40, 805–830. 10.1017/S0272263118000037

[B89] Zuo M. Hu Y. Luo H. Ouyang H. Zhang Y. (2022). K-12 students' online learning motivation in China: an integrated model based on community of inquiry and technology acceptance theory. Educ. Inform. Technol. 27, 4599–4620. 10.1007/s10639-021-10791-x 34754268 PMC8568678

